# Integration of root phenes for soil resource acquisition

**DOI:** 10.3389/fpls.2013.00355

**Published:** 2013-09-12

**Authors:** Larry M. York, Eric A. Nord, Jonathan P. Lynch

**Affiliations:** ^1^Intercollege Program in Ecology, The Pennsylvania State University, University ParkPA, USA; ^2^Department of Plant Science, The Pennsylvania State University, University ParkPA, USA

**Keywords:** root architecture, phenomics, functional traits, ideotype, soil resources

## Abstract

Suboptimal availability of water and nutrients is a primary limitation to plant growth in terrestrial ecosystems. The acquisition of soil resources by plant roots is therefore an important component of plant fitness and agricultural productivity. Plant root systems comprise a set of phenes, or traits, that interact. Phenes are the units of the plant phenotype, and phene states represent the variation in form and function a particular phene may take. Root phenes can be classified as affecting resource acquisition or utilization, influencing acquisition through exploration or exploitation, and in being metabolically influential or neutral. These classifications determine how one phene will interact with another phene, whether through foraging mechanisms or metabolic economics. Phenes that influence one another through foraging mechanisms are likely to operate within a phene module, a group of interacting phenes, that may be co-selected. Examples of root phene interactions discussed are: (1) root hair length × root hair density, (2) lateral branching × root cortical aerenchyma (RCA), (3) adventitious root number × adventitious root respiration and basal root growth angle (BRGA), (4) nodal root number × RCA, and (5) BRGA × root hair length and density. Progress in the study of phenes and phene interactions will be facilitated by employing simulation modeling and near-isophenic lines that allow the study of specific phenes and phene combinations within a common phenotypic background. Developing a robust understanding of the phenome at the organismal level will require new lines of inquiry into how phenotypic integration influences plant function in diverse environments. A better understanding of how root phenes interact to affect soil resource acquisition will be an important tool in the breeding of crops with superior stress tolerance and reduced dependence on intensive use of inputs.

## INTRODUCTION

Global food security is a serious challenge (Funk and Brown, 2009), with approximately 870 million people experiencing chronic undernourishment (FAO et al., 2012). In much of the developing world, use of nitrogen (N) and phosphorus (P) fertilizers is relatively low, leading to substantial reductions in crop yields (FAO, 2008). In developed nations intensive use of fertilizers is associated with greater crop yields (Roberts, 2009). However, crop plants in these agricultural systems take up only a portion of the applied nitrogen fertilizer (Goulding, 2000), and the remainder pollutes water and the atmosphere (Jenkinson, 2001). Furthermore, phosphorus fertilizers are a non-renewable resource, and global production of phosphorus is expected to peak around the year 2033 (Cordell et al., 2009). Increasing crop acquisition of both nitrogen and phosphorus is therefore a desirable goal for both subsistence and commercial agriculture. Belowground properties of natural ecosystems are also receiving attention because of their influence on important processes including carbon sequestration (Eissenstat et al., 2000) and community structure (Craine et al., 2002).

Root architecture, the spatial arrangement of a root system, has been shown to be important in agricultural systems (Lynch, 1995; Ho and Lynch, 2004; Hirel et al., 2007) and natural systems (Mahall and Callaway, 1992; Comas and Eissenstat, 2009) for nutrient acquisition, plant interactions, and nutrient cycling. Understanding the contribution of specific root traits, or phenes, to root system function is critical for crop improvement because it allows identification of traits that contribute desired functions (Kell, 2011; Lynch and Brown, 2012). High-throughput root phenotyping is an important tool in this context as it permits the profiling of the extent, magnitude, and distribution of root traits in crop germplasm, and because phenotyping is limiting progress in crop breeding (Furbank and Tester, 2011). Advances in high-throughput phenotyping of roots (Grift et al., 2011; Trachsel et al., 2011; Zhu et al., 2011) will enable focused efforts to improve crop nutrient acquisition by selection for root ideotypes and to understand the influence of inter-and-intraspecific root system variation on community structure and ecosystem function.

Ideotype, or trait-based, breeding was proposed by Donald (1968) as a way to combine traits that would each contribute to increased yield. He identified a flaw in “deficit elimination” or “selection for yield” approaches in that they do not seek to answer *how* increased yield is created (Donald, 1962). Instead, he proposed studying traits in isolation to understand how they contribute to yield then combining such yield improving traits through traditional breeding. Crop breeding programs commonly combine traits, especially in the pyramiding of traits associated with disease resistance (Shen et al., 2001; Singh et al., 2001; Steele et al., 2006). This approach has contributed substantially to yield gains in several crops, including maize, wheat, and common bean (Mock and Pearce, 1975; Kelly and Adams, 1987; Reynolds et al., 1994; McClean et al., 2011). The trait-based approach inherent in the concept of ideotype breeding forced researchers to not only consider traits of interest in isolation, but also to consider relationships among traits. This is illustrated by the work of Rasmusson (1987), demonstrating that compensation among plant organs can lead to tradeoffs, such as increasing head numbers being associated with fewer, smaller kernels in barley. The integration of traits determines how the whole plant functions and remains an underutilized aspect of ideotype breeding.

A body of work on phenotypic integration in the field of evolutionary biology and ecology has also considered some aspects of the relationships among traits (Murren, 2002; Pigliucci, 2003). In this context phenotypic integration has been defined as the “pattern of functional, developmental, and/or genetic correlation (however measured) among different traits in a given organism” (Pigliucci, 2003). In plants, this area of research originated with the work of Berg (1960) who identified clusters of correlated traits. Strong correlations between traits could imply shared functions, with correlations among traits possibly maintained by stabilizing selection. In some cases researchers have focused on how groups of correlated traits affect plant function in specific ecological contexts (Lechowicz and Pierre, 1988). Economic spectrums that relate traits by their costs and functions have been identified in leaves (Wright et al., 2004), and proposed for roots, though evidence for a root economic spectrum remains inconclusive (Chen et al., 2013). In this research, phenotypic diversity within species or populations has typically been viewed as noise rather than as an important response to heterogeneous and unpredictable environments, competition, and phenotypic plasticity. Both ecological and agricultural research have converged upon concepts of integration through genetic, physiological, and developmental correlation (Grafius, 1978), though researchers in both areas seem to be largely unaware of the other.

Trait “stacking” in genetically modified crops (GMCs) is another form of ideotype breeding and trait integration. Traits of interest here are usually of the “deficit elimination” type, such as reducing susceptibility to insects or herbicides. First-generation stacks included *Bt* toxin-producing and glycophosphate-resistant GMCs that were introduced in 1998 (James, 2000). In order to decrease the selection for *Bt* toxin resistance in agricultural insect pests, 2nd-generation stacks combine several modes of actions for the same trait, which also reduces requirements for non-GMC refuge areas (Que et al., 2010). Stacking technologies have rapidly developed to higher numbers of combined traits, such as the nine foreign proteins combined in *SmartStax*^TM^ (Marra et al., 2010). Gene stacking does lead to trait interactions in that most GM traits enhance growth in some situations, and combining modes of action decreases the ability of pests to adapt. Trait synergisms have been considered by biotechnology companies (Then, 2011), but only in terms of multiple modes of action for pest control, similar to the pyramiding of genes for disease resistance through introgression breeding.

Traditional plant breeding has attempted to combine traits that are helpful in isolation, and transgenic crops have also made progress in the stacking of particular traits. Ecologists have observed correlations among traits and between traits and plant function. However, our understanding of non-additive trait interactions is limited, and this is particularly true in root biology. Here we propose a theoretical framework for evaluating root system phenes and their functional interactions in the context of soil resource acquisition. We will show that the combining of traits does not always lead to a simple accumulation of additive effects, so plant biologists and breeders must take into account trait synergisms.

## THEORETICAL FRAMEWORK

### WHAT IS A PHENE?

**FIGURE 1:**
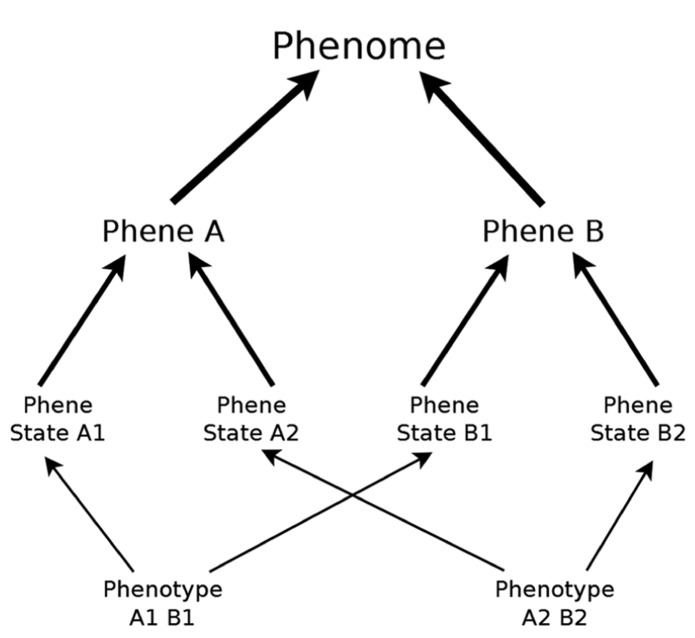
**Studying the characteristics of phenotypes of different individuals allows us to identify phenes and their existent states.** The phenome is the total possible phenotypic potential of a taxon, including all possible phene states. The phenotypes presented here do not represent all possible phenotypes of this phenome.

“Phene” was used as early as 1925 in animal genetics to describe phenotypic traits under genetic control (Serebrovsky, 1925), and has been used extensively in European and Russian agricultural literature (e.g., Gustafsson et al., 1977). Phene can be defined concisely:* phene* is to *phenotype* as *gene* is to *genotype* (Lynch, 2011; Pieruschka and Poorter, 2012). Just as genes have variants called alleles, phenes have variants we will refer to as *phene states* (*phene* is to *trait* as *phene state* is to *attribute*). The particular combination of states for all phenes constitutes the phenotype of an individual organism. We will use *phenome* as the totality of all possible phene states of a taxon, i.e., phenotypic potential (**Figure 1**). Alternative more generic terms such as *traits*, *characters*, and *attributes* have been used with ambiguity that can lead to confusion (Violle et al., 2007), such as by referring to properties at several levels of biological organization or by using trait to refer to either phenes or phene states. Lynch and Brown (2012) proposed that the most useful and meaningful phenes are *elementary* and *unique* at their level of biological organization (e.g., organ, tissue, cell). For example, an elementary root architectural phene should not be the product or aggregation of other more basic architectural phenes. The genetic and developmental processes giving rise to phenes should be unique, i.e., a phene is elemental because it has a unique developmental pathway. Some phenes may be under single gene control, and have phene states that are discrete. Many phenes are probably quantitative traits resulting from the interaction of many genes and the environment, and will show a continuous distribution of phene states. Many measurements of plant phenotypes are aggregates of multiple elemental phenes. For example, rooting depth has been shown to be influenced by separate phenes, such as root growth angle (Trachsel et al., 2013) and aerenchyma (Zhu et al., 2010a). Such plant characteristics may be referred to as *phene aggregates*. Plant measurements similar to yield, plant mass, or nutrient content will not be referred to as phenes or as phene aggregates. Rather, they are functional responses dependent on the state of many components of the plant phenotype.

**FIGURE 2:**
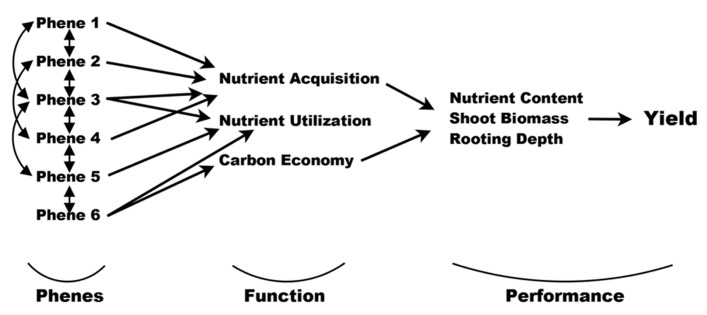
**Phenes and their interactions influence plant functions such as nutrient acquisition, utilization, and carbon economy.** In turn, these functions affect agricultural performance measures such as shoot biomass and nutrient content. Ultimately, all these lead to yield (or fitness). Yield is far removed from base functions, which themselves can be multi-tiered and reciprocating. The original diagram was made by Arnold (1983) and reworked for plant ecology by Violle et al. (2007). Here we present it for a phene-centric view in agriculture.

**FIGURE 3:**
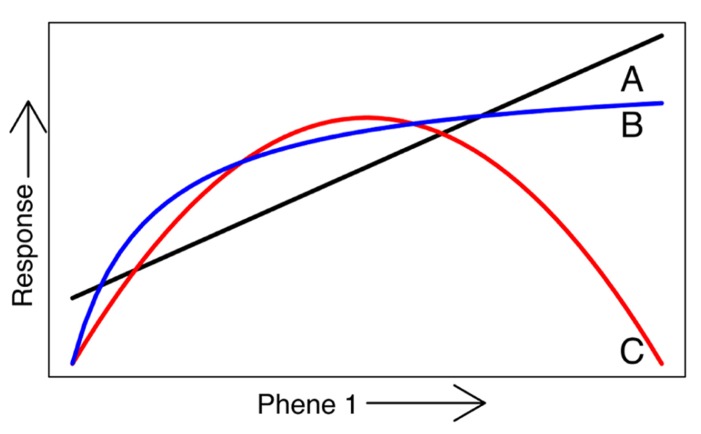
**A phene-function response curve shows the influence of a single continually varying phene on a plant functional response.** A phene may have a linear effect on the response **(A)**, asymptote **(B)**, or have an optimum at middle states **(C)**.

Phene states make up phenotypes, which are individual manifestations of the phenome of a species. The root phenes of interest to us here have functional utility for resource acquisition (Lynch, 2011), and are components of root architecture, morphology, anatomy, or physiology. In turn, these functions influence agricultural performance such as biomass and yield, or plant fitness in natural systems (**Figure 2**), *sensu* Arnold (1983) and Violle et al. (2007). Functional utility can be assessed by comparing the functional responses of different phene states. For example, it has been shown that plants with longer root hairs acquire more phosphorus than plants with shorter root hairs or none at all (Bates and Lynch, 2000; Yan et al., 2004; Zhu et al., 2010b). The comparison of the phosphorus acquisition responses of these two root hair phene states demonstrates that the root hair length phene is important for P acquisition, with longer root hairs leading to greater P acquisition. A phene-function response curve shows the influence of a single continually varying phene on a plant function (**Figure 3**).

### ROOT PHENE CLASSIFICATION

#### Root phenes classified by function, foraging strategy, and metabolic influence

**FIGURE 4:**
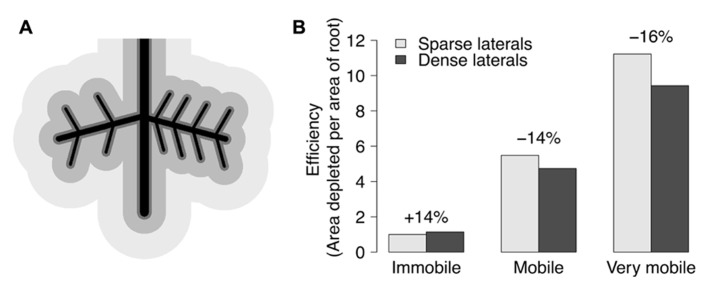
**(A)** Black lines depict a simplified root system with a lateral root on each side of a tap root. The left side has 4 second order laterals, while the right side has 8 second order laterals. The darkest gray area around roots depicts the depletion zone of immobile resources (like P), while the medium gray depicts the depletion zone of mobile resources (like N), and the lightest gray represents very mobile resources (like water). **(B)** Efficiency is shown by the quotient of the area (pixel counts) of a respective resource’s depletion zone divided by the area of the roots for each half of the root system with sparse or dense second order laterals. Dense laterals increase the efficiency for an immobile resource, but decrease efficiency for mobile resources. Differences would be inflated if areas were converted to volumes.

Phenes can be classified in numerous ways. A mechanistic classification of root phenes can be made on the basis of whether they primarily affect resource *acquisition *or resource *utilization.* Phenes that affect soil resource acquisition generally affect the coincidence of root foraging and soil resource availability in time and space. Phenes that affect resource utilization influence how efficiently resources are used for plant functions including growth, further resource acquisition, and reproduction. Phenes that affect resource acquisition can be further classified based on foraging strategy. Foraging strategies exist along a continuum from phenes that influence soil exploration to those that influence soil exploitation. Exploration phenes influence the spatial and temporal exploration of soil domains by roots and root symbionts. Exploitation of soil resources describes how thoroughly resources are acquired within a given soil domain, i.e., with no further soil exploration. Fitter proposed a measurement of acquisition efficiency to be the quotient of soil volume depleted to total root system volume (Fitter et al., 1991). This volume depends on the mobility of nutrients. Phosphorus depletion zones are only a few millimeters in diameter, while those for nitrate may be 10–100 times larger due to the 1000-fold difference between phosphate and nitrate in effective diffusion coefficients (Barber, 1984). A phene state can increase exploration for an immobile resource by entering new soil domains, while also increasing the exploitation of a domain for a more mobile resource by increasing the intensity of its acquisition (**Figure 4**). The differences in mobility between mobile and immobile nutrients give rise to the *root system depletion zone* and *root surface depletion zones *(lighter gray vs. dark gray in **Figure 4**), respectively (Bray, 1954). The growth angle of axial roots (e.g., nodal roots in maize, basal roots in common bean) influences the relative exploration of shallow and deep soil domains. Topsoil foraging has been shown to be important for phosphorus acquisition in both maize and common bean (Lynch and Brown, 2008), while deep soil foraging has been proposed to be important for the acquisition of water and nitrate (Lynch, 2013). Exploitation phenes affect the rate of nutrient uptake by increasing root density (number or length of roots in a volume) through greater numbers of axial roots, lateral branching, or root hairs and rhizosphere modification, for example. Rhizosphere modification includes decreasing the pH by releasing protons, organic acids, and by exudation of enzymes that release phosphorus from organic compounds (Lambers et al., 2006). Mycorrhizal symbioses can affect both exploration and exploitation, depending on the spatial scale and resource. Mycorrhizal fungi increase soil exploration by the growth of their hyphae, and exchange phosphorus for carbon with their host plant (Harley, 1989). Resource acquisition phenes not only differ in foraging strategies but in how they influence plant metabolism, and effects on metabolism are the mechanism for utilization phenes.

The functional utility of root phenes for soil resource acquisition is strongly influenced by rhizoeconomics (Lynch and Ho, 2005; Lynch, 2007a), i.e., their relative costs and benefits. One of the major costs of roots is their metabolic demand. Several economic currencies can be used to estimate cost/benefit relationships, such as carbon, nitrogen, and phosphorus (Lynch and Rodriguez, 1994; Lynch and Beebe, 1995). Metabolic costs can be partitioned into construction and maintenance costs (Chapin et al., 1987). Root construction costs are generally strongly influenced by root volume which is proportional to length and diameter, so phenes which determine these (e.g., elongation rate, branching, number of roots formed, and root diameters) will influence construction costs. Roots, like all plant tissues, require not only carbon, but also mineral nutrients for construction and maintenance. Phenes have been identified that alter root metabolic demand. “Root etiolation,” or decreasing diameter in order to increase length, has been proposed as an adaptive trait for nutrient acquisition (Lynch and Brown, 2008), with empirical support provided in maize (Zhu and Lynch, 2004). Root cortical aerenchyma (RCA) converts living cortical tissue to air space via programmed cell death. This lowers the respiration of root segments (Fan et al., 2003), and has the additional benefit of mobilizing nutrients for other uses (Postma and Lynch, 2010). An economic classification of root phenes is based on how they influence metabolism. **Table 1** presents a number of root phenes and their classification according to these three schemes (acquisition vs. utilization, exploration vs. exploitation, and metabolic influence vs. no metabolic influence).

**Table 1:** Classification of root phenes.

Root phene	Mechanism	Foraging	Economy
**Axial root growth angle**	Acquisition	Exploration	Neutral
**Root growth rate**	Acquisition	Exploration	Influencing
**Number of axial roots**	Acquisition	Exploration	Influencing
		Exploitation	
**Lateral root branching**	Acquisition	Exploitation (N)	Influencing
		Exploration (P)	
**Root hair density**	Acquisition	Exploitation (P)	Neutral
**Root hair length**	Acquisition	Exploitation (P)	Neutral
**Rhizosphere modification**	Acquisition	Exploitation (P)	Influencing
**Aerenchyma**	Utilization		Influencing
**Root etiolation**	Utilization		Influencing

*Classification of a particular root phene begins by determining its mechanism affecting resource uses, acquisition or utilization. Resource acquisition phenes are classified based on their foraging strategy, exploration or exploitation for a particular resource, with nitrogen (N) representing mobile and phosphorus (P) representing immobile resources. All root phenes are classified by whether they influence metabolic economy or are neutral.*

#### Not all root measurements are root phenes

An array of root measurements are commonly made in both agricultural and natural systems that do not meet the definition of an elemental phene. Rather, most of these root measurements represent phene aggregates that are influenced by the states of several root phenes (**Table 2**). Others, such as total root length, are *functional responses* that are influenced by states of phenes through their influence on soil resource acquisition and eventual photosynthate allocation to the root system. Unexplained variation in these measurements may be resolved by more thorough documentation of constituent root architectural, anatomical, and physiological phenes. These measurements may often be referred to as traits, which highlights the difference between the common usage of “trait” and the biological definition of “phene”.

**Table 2:** Relation of root measurements to root phenes.

Root measurement	Definition	Influential phenes	Reference
**Total root length**	The cumulative length of an entire plant root system (m) that is partially a *functional response*.	Axial root length, number of axial roots, lateral branching, lateral length	Zhu et al. (2006), Brun et al. (2010)
**Root length density**	The cumulative length of roots per some volume, often from soil cores or monoliths (m cm^-^^3^) that is a *phene aggregate* dependent on the states of constituent phenes.	Axial root length, number of axial roots, lateral branching, lateral length	Ho et al. (2005), Miguel et al. (2013)
**Specific root length**	The root length per unit mass (m g^-^^1^) that is a *phene aggregate* dependent on the states of constituent phenes.	Xylem area, phloem area, number of cortical cells, cortical cell size, aerenchyma area, secondary development	Fan et al. (2003), Jaramillo et al. (2013)
**Root tissue density**	The mass of roots per unit root volume (g cm^-^^3^) that is a *phene aggregate* dependent on the states of constituent phenes.	Xylem area, phloem area, number of cortical cells, aerenchyma area, secondary development	Fan et al. (2003), Jaramillo et al. (2013)
**Rooting depth**	The deepest depth at which roots from a plant are observed (m). Alternatively, the depth at which 95% of root length is at or above may be used. Both are phene aggregates dependent on the states of constituent phenes, and will be influenced by total root length.	Axial root angles, axial root length, number of axial roots, lateral branching, lateral length	Ho et al. (2005), Miguel et al. (2013)
**Root respiration**	The rate of CO_2_production due to root metabolism (mmol CO_2_ m^-^^1^ root s^-^^1^) that is a *phene aggregate* dependent on the states of influencing phenes and their contributions to total root segment respiration.	Number of cortical cells, cortical cell size, aerenchyma area, N content, secondary development	Fan et al. (2003), Zhu et al. (2010a), Jaramillo et al. (2013)
**Root longevity**	The length of time between the formation and loss of a root (s) that is a *functional response* dependent on root defenses and stress physiology.	Phenolic concentrations, lignin concentration, number of cortical cells, cortical cell size, aerenchyma area	Eissenstat et al. (2000)
**Topological index and fractal dimension**	Ratios of different measures summarizing the complexity of a network (unitless) that are *phene aggregates* dependent on the states of constituent phenes.	Axial root length, number of axial roots, lateral branching, lateral length	Fitter and Stickland (1992),Walk et al. (2004)

*Many common measures of root system and individual root properties are examples of phene aggregates that are influenced by several more elemental root phenes, and some are partially functional responses dependent on plant performance. These root measures are defined and the phenes that influence the measure are listed. Here, lateral branching includes the branching of successive orders of laterals, i.e., including laterals of axial roots, laterals of laterals, etc.*

### HOW DO PHENES INTERACT?

#### Functional response interactions

**FIGURE 5:**
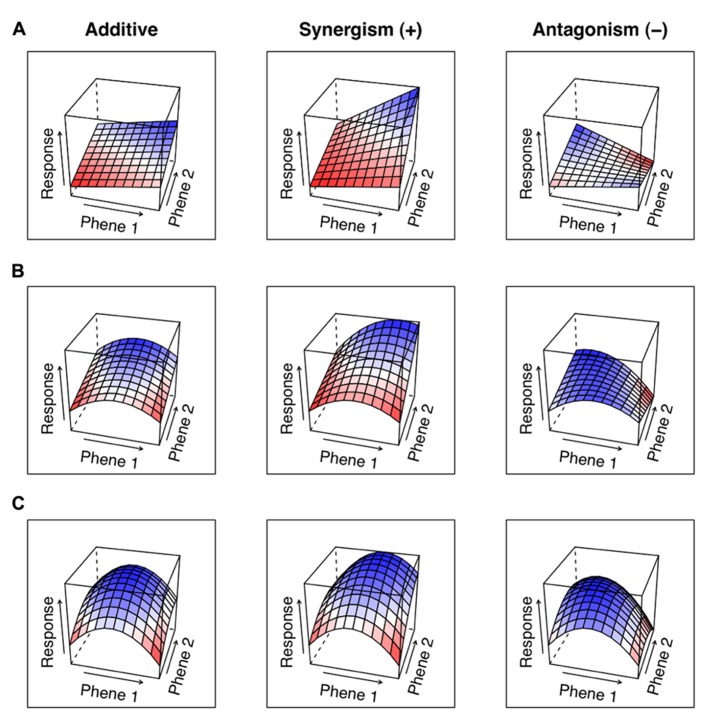
Panel **(A)** shows the functional response landscape of two phenes that have linear effects in isolation. Panel **(B)** shows one phene with a linear effect and one with a central optimum. Panel **(C)** shows two phenes with optimums at middle phene states. Synergisms are shown by responses greater than the additive, while antagonistic effects are shown as being less than the additive.

The utility of a phene can be assessed by comparing the functional responses of varying states of the phene. Similarly, the interaction of two phenes can be assessed by combining at least two phenes states of two different phenes and measuring the functional response of each combination. In such a situation, the null hypothesis is that the functional response of two phene states from two different phenes will be additive. The particular phene state combination is synergetic when the functional response exceeds the sum of the responses of the phene states in isolation. Antagonistic interactions occur when the functional response of phene states in combination is worse than that expected from the sum of their responses in isolation. We can describe the mechanistic basis of the interaction based on the classifications of the component phenes. A phene-function response landscape graphically demonstrates how the simultaneous changes of two or more phenes affect a function (**Figure 5**).

#### Foraging strategy interactions

Phenes interact through their effects on *foraging* when the mechanism through which one phene affects foraging directly interacts with the mechanism of another phene affecting foraging. For example, axial roots with shallow growth angles will increase the exploration of soil with greater amounts of phosphorus, while increased root hairs will increase the exploitation of the explored soil. The combined states of shallow growth angles and increased root hairs may have a synergetic interaction beyond what would be expected based on their additive effects on phosphorus acquisition (see Case Study 2).

#### Economic interactions

The *economic *interaction of two phenes is mediated by the metabolic budget of the plant. Two metabolism influencing phenes will exhibit tradeoffs when occupying more metabolically demanding states. These tradeoffs are expected between root classes, or even between number and length within a class (Walk et al., 2006; Rubio and Lynch, 2007). Building more of one type of root will necessarily limit the metabolic resources available for building other types, or decrease the resources available for elongation of existing roots. However, feedbacks between nutrient acquisition and increased photosynthesis that allow further root growth are possible. Conversely, a metabolically neutral phene will have no economic interaction with a metabolism influencing phene.

#### Phene modules

Combinations of specific phene states may be more likely to be found together in individuals of a taxon when they act as a functional module through foraging and economic interactions. Modules are aggregates of components that are related, such as in the context of molecular pathways (Hartwell et al., 1999), architectural modules such as leaves, flowers, and roots, and even entire plants as modules in an ecosystem (Prusinkiewicz, 2004). One useful definition for module in the context of phene interactions is a group of phenes that behave synergistically. In roots, such functional module components probably belong to the same parent root class, similar to the “modular unit” suggested by Pregitzer et al. (2002) as lateral branches of tree roots consisting of several orders of the finest roots. In crops such as common bean and maize, these modules are initiated from and include the major axes, i.e., basal roots in bean, nodal roots in maize. Foraging interactions are more likely to occur in modules composed of phenes that are close together because their likelihood of coinciding with a soil resource increases.

#### Environmental interactions

It is well known that the abiotic and biotic environments can affect the phene states of an organism through the phenomenon of phenotypic plasticity (West-Eberhard, 1989; Callaway et al., 2003). For example, roots have been observed to proliferate in patches of nutrients (Drew and Saker, 1975; Granato and Raper, 1989), change rooting angle (Bonser et al., 1996), change root hair density (Ma et al., 2001a), and alter axial elongation and lateral root density in response to phosphorus availability (Borch et al., 1999). Root phenotypic plasticity constitutes one type of phene–environment interaction. Another type is based on tradeoffs and synergies that may exist between root phenes and particular soil resources, i.e., phene × environment × functional response interactions. For example, in low phosphorus soils, phenotypes with shallow root growth angles perform better than phenotypes with steep root growth angles, but in high phosphorus conditions both perform equally well. Steep-angled phenotypes are better at acquiring water during terminal drought (Ho et al., 2005), so there is an architectural tradeoff for root growth angle for acquiring resources at different depths in the soil. When both phosphorus stress and terminal drought occur together, shallow-rooted phenotypes performed better because early P uptake allowed the growth of more extensive root systems that then conferred greater tolerance to the terminal drought. Phene × phene × environment interactions are more complicated than single phene × environment interactions, but must be studied in order to understand how plants cope with multiple stresses, and how suites of traits influence fitness.**

#### Interplant interactions

Root competition among plants of different species plays an important role in shaping plant communities (Schenk, 2006) and in the performance of interspecific polycultures in agriculture (Wilson, 1988; Postma and Lynch, 2012). Competition is expected to be greater for mobile nutrients than relatively immobile nutrients (Postma and Lynch, 2012; Wilberts et al., 2013). Little is known about how specific root traits affect competition and facilitation, but there are a few examples. *Arabidopsis* wildtypes with root hairs were shown to have a competitive advantage over root hairless *rhd2* mutants in low phosphorus media (Bates and Lynch, 2001). Similarly, *Arabidopsis* wildtypes out-competed *axr4* mutants with decreased numbers of lateral roots in low phosphorus, but not in low nitrogen (Fitter et al., 2002). Architectural multilines of common bean composed of equal portions of plants with shallow and steep basal root angles had Land Equivalent Ratios greater than unity (Henry et al., 2010), which means more area must be planted of the monocultures in order to achieve the same levels of yield as the multilines. This implies a competitive release of the dominant shallow-rooted plants when grown with steep-rooted plants in low phosphorus soils. Common beans were shown to alter root architecture in the presence of neighboring plants due to localized phosphorus depletion (Nord et al., 2011). Clearly, understanding phenes requires an understanding of how phenes will react to other phenes, the environment, and other plants.

#### Phene integration

Foraging, economic, environmental, and interplant interactions of phenes create an integrated phenotype. The integrated phenotype is more than simply a collection of isolated traits, but rather is a suite of interacting phenes that affect plant functions. These interactions cannot simply be assumed to be additive and will depend on the environmental context. Phene integration occurs at all levels of phenotypic organization, from cells, to modules, to the whole plant.

**FIGURE 6:**
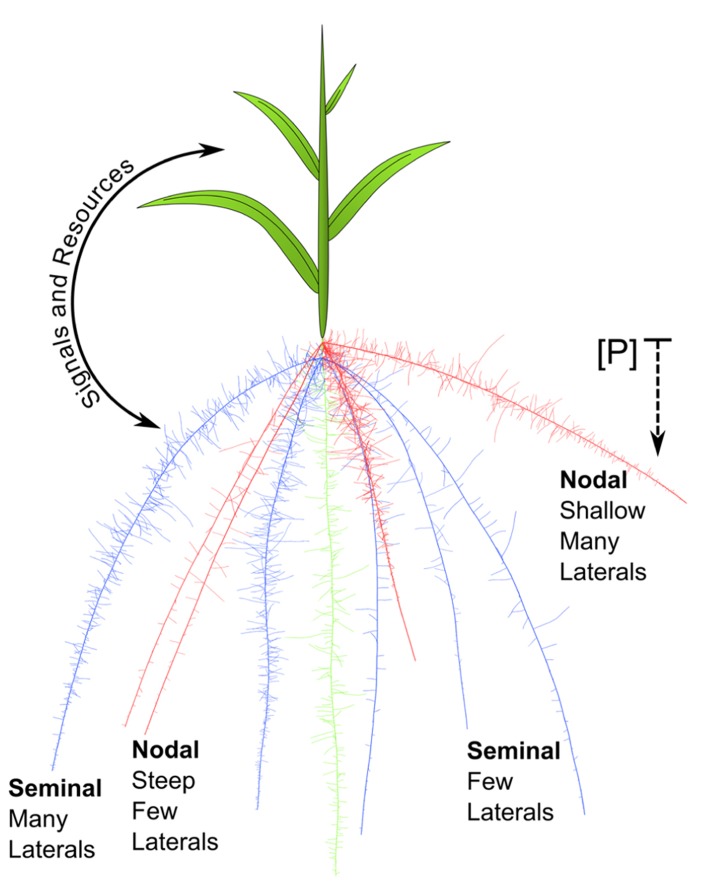
**A maize seedling is depicted.** Seminal roots (blue) and primary root (green) emerge from the seed. One whorl of nodal roots (red) is shown emerging from belowground stem tissue. The nodal roots on the left have steep growth angles, while those on the right are shallow. The shallow nodal roots on the right also have dense laterals, while the steep nodal roots on the left have sparse laterals. In the context of phosphorus acquisition from the epipedon, shallow nodal roots with many laterals will have a synergistic interaction because they are acting within the same module. Though the seminal roots on the left have many laterals they will not interact synergistically for foraging with nodal root traits because they are in a different root class module. The whole plant is integrated by reciprocal signaling between shoot and roots and by balancing the production of photosynthates with soil resource acquisition.

Phenes may interact via resource partitioning and signaling, even between roots and shoots. Typically, shoots provide photosynthates to the roots, while roots supply soil resources to the shoot. Thornley (1972) developed a mathematical model with two pools, shoot and roots, and two substrates, carbon and nitrogen, which are supplied by the shoot and roots, respectively. This simple source–sink model demonstrated that plants should balance shoot and root activity and invest in the organs that produce the most limiting resource, and continues to guide whole plant modeling. Empirical work demonstrates that aboveground and belowground organs communicate their internal and environmental status to each other in order to integrate plant function in dynamic environments. For example, root ABA signals induce stomatal closure in leaves which decreases transpiration (Davies and Zhang, 1991). The plant shoot is partially responsible for perceiving the internal nitrogen status and uses reduced nitrogen compounds and auxin to signal roots to form lateral roots (Ruffel et al., 2011). Interestingly, roots can also influence shoot branching through auxin signaling (Bennett et al., 2006), which might suggest root perception of the soil environment informs the regulation of shoot growth. These interactions suggest that another form of phene interaction may be information exchange, which may apply within the root system as well. The global leaf economic spectrum demonstrates that leaves from a variety of species representing diverse functional groups are constrained by development and natural selection to fall along a single spectrum for a variety of traits (Wright et al., 2004). A direct interaction between a shoot phene such as leaf morphology and an RSA phene like lateral branching is unlikely. Rather, the shoot and root organs integrate information processing and metabolism, and balance production of photosynthates with acquisition of soil resources (**Figure 6**).

#### Hypotheses regarding the integration of root phenes

We propose the following hypotheses regarding the integration of root phenes:

(1) Functional synergisms will occur among foraging phenes that act within a module including the axial root and its subordinate roots.
(2) Functional synergism will increase as the number of positively acting phene states combined is increased.
(3) Metabolic tradeoffs will limit synergism created by combining foraging phene states that demand more metabolic resources, except when alleviated by phenes in states that relieve metabolic constraints.
(4) Synergisms will be more likely to occur when combining metabolically neutral phenes in positively acting states.
(5) The large diversity of root system phenotypes, i.e., the particular combination of phene states of an individual, is partially explained by the interactions of phenes within plants, between plants, and between phenes and the environment.

## CASE STUDIES

Research on phene interactions is nascent, and this is especially true in the case of roots. Much of the evidence for root phene integration comes from research with *SimRoot*, a functional–structural plant model focusing on root system architecture and nutrient acquisition (Lynch et al., 1997; Postma and Lynch, 2010), though we will also discuss empirical evidence and experimental approaches for studying phene interactions.

### ROOT HAIR LENGTH × ROOT HAIR DENSITY

Root hairs are subcellular extensions of root epidermal cells that are particularly important for the acquisition of immobile nutrients such as phosphorus. Root hairs can vary in density (i.e., number of root hairs per unit root surface area) and in length. Diversity for both of these traits is evident in several species including common bean, soybean, and maize (Wang et al., 2004; Yan et al., 2004; Zhu et al., 2005a). *SimRoot *was employed to test interactions among root hair length, root hair density, proximity of root hair appearance to the apical meristem, and the spatial patterning of hair-bearing cells (trichoblasts) and non-hair-bearing cells (atrichoblasts) in *Arabidopsis* (Ma et al., 2001b). The synergetic effect of increased root hair length and density (RHLD) phene states was 272% greater than their expected additive effects. Root hair formation nearer the root tip increases P acquisition, while number of files had positive effects when more numerous. All positive phene states were compared to their expected additive function response in two-way, three-way, and four-way combinations. On average, synergetic effects increased with the number of positive interactions: two-way, 168%; three-way, 232%; and four-way, 371% greater than additive effects (new calculations from original data). Changing RHLD in *Arabidopsis* had no direct effect on root respiration (Bates and Lynch, 2000). We hypothesize that metabolically neutral phenes will have the greatest synergisms because of the lack of economic tradeoffs. As this example shows, the magnitude of phene synergisms may increase with the number of positively interacting phene states (Hypothesis 2).

### LATERAL BRANCHING × ROOT CORTICAL AERENCHYMA

Variation for lateral root length and density has been observed in both the primary root and nodal roots of maize (Zhu et al., 2005b; Trachsel et al., 2011). Greater lateral root length and density would permit greater soil exploration, and so would improve acquisition of soil resources. However, increased lateral branching has high metabolic demand, and due to competing sinks it could influence the growth of other root classes. This trade-off could be alleviated by decreasing metabolic demand in other ways. *SimRoot* was used to test the hypotheses that increased lateral root branching would increase N and P acquisition and that this phene would be affected by the formation of aerenchyma (Postma and Lynch, 2011). At the lowest level of nitrogen, there was a 42% reduction in shoot dry weight compared to the expected additive effects of increasing lateral root branching and forming aerenchyma, which constitutes a functional antagonism. However, at the intermediate level of nitrogen a synergetic interaction 220% greater than the expected additive effects was observed. In the low phosphorus condition, the synergetic interaction was 33% greater than the expected additive effects. This broad range of interaction demonstrates the importance of environmental context.

### ADVENTITIOUS ROOT NUMBER × ADVENTITIOUS ROOT RESPIRATION AND BASAL ROOT GROWTH ANGLE

Adventitious roots emerge from the hypocotyl in common bean (*Phaselous vulgaris*) and have less construction and maintenance costs than basal roots (Miller et al., 2003). Adventitious roots emerge in the topsoil and typically have extremely shallow growth angles, so they were hypothesized to be an adaptive trait for topsoil foraging. Basal roots are the principal axial roots in common bean, and a shallow growth angle for basal roots has been shown to be important for topsoil foraging (Bonser et al., 1996; Liao et al., 2004; Ho et al., 2005; Henry et al., 2010). Adventitious roots were found to have a range of respiration rates from the same as tap roots, to 400% greater than tap roots (Bouma et al., 1997; Walk et al., 2006). Because phosphorus has low soil mobility, it accumulates in the topsoil from the deposition of senesced plant tissue (Anderson, 1988). Both functional response and economic interactions were expected between adventitious root number (ARN) and adventitious root respiration (ARR), and between ARN and basal root growth angle (BRGA), which was tested in *SimRoot* (Walk et al., 2006). Increasing ARN greatly increased phosphorus acquisition when ARR was the same as tap root respiration, and marginally benefited phosphorus acquisition when ARR was two times tap root respiration. When ARR was four times greater than tap root respiration, there was a negative relation between increasing ARN and phosphorus acquisition. At the highest level of ARR, not enough metabolic resources were available for the construction of root length adequate for phosphorus acquisition. This shows a functional response antagonism between greater states of ARN and ARR that is mediated through an economic interaction. ARN was also expected to interact with BRGA. However, only additive effects were observed between greater ARN and more shallow BRGA, which suggests adventitious roots and basal roots function as independent modules (Hypothesis 1).

### NODAL ROOT NUMBER × ROOT CORTICAL AERENCHYMA

**FIGURE 7:**
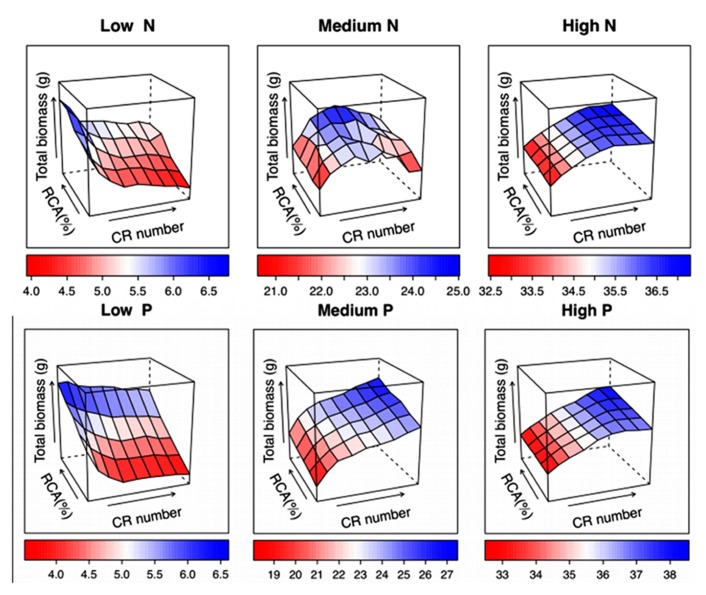
**Phene integration of root cortical aerenchyma (RCA) and crown root (CR) number was studied in maize using SimRoot across a range of nitrogen (N) and phosphorus (P) levels.** These simulation results demonstrate linear, asymptotic, and optimum single phene responses and their interactions.

Unpublished results from *SimRoot* show interaction between RCA and number of nodal roots in maize (**Figure 7**). Across a range of N and P availability, root length and total biomass were strongly affected by nodal root number. RCA had little to no effect on biomass or root length when there were fewer than optimal crown roots, but increased root length and biomass with optimal or greater than optimal numbers of nodal roots, especially with suboptimal N or P. Because optimal nodal root number differed between N deficient and P deficient conditions, the range of nodal root numbers where RCA increased biomass depended on the environment.**At medium levels of nitrogen and phosphorus, the synergetic effects of greater numbers of crown roots and RCA were 31.6% and 132% greater than the expected additive effects, respectively.

### BASAL ROOT GROWTH ANGLE × ROOT HAIR LENGTH AND DENSITY

**FIGURE 8:**
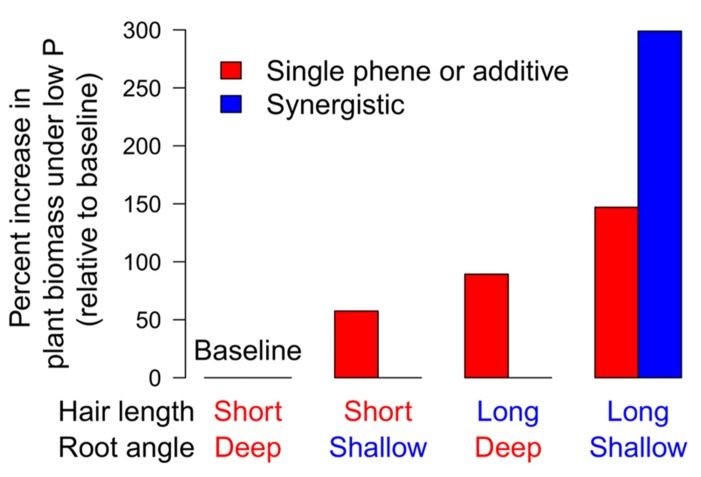
**Long root hairs and shallow basal root angles interact synergistically on phosphorus acquisition in the field (created from Miguel, 2012)**.

In common bean, BRGA is a soil exploration phene and was hypothesized to influence the utility of the root hair phene, which affects exploitation, by determining the placement of root hairs in the soil profile. A field study was conducted in Mozambique, comparing three recombinant inbred lines (RILs) for each of four phenotypes representing all combinations of shallow and deep BRGA and low and high RHLD; Miguel, 2012). In low P soil, shallow BRGA increased shoot growth by 57.7%, and greater RHLD increased shoot growth by 89.3% (**Figure 8**). Shoot mass of the combined positive states (shallow angle and greater RHLD) was 298% greater than the base line (steep angle and lower RHLD), which is twice the expected additive effect. Root hairs along with the basal roots or basal root laterals on which they form constitute a functional module which gives rise to high levels of synergism (Hypotheses 1 and 4).

### EVIDENCE FOR ROOT PHENE FUNCTION AND INTERACTION IN NATURAL DOMAINS

Variation in root phenes has been observed among wild species along with correlation between phenes, such as between specific root length and lateral branching (Comas and Eissenstat, 2009). Differences in rooting depth among grassland species has been proposed as one contribution to the relationship between biodiversity and ecosystem productivity by allowing plants to exploit particular soil niches (Fargione and Tilman, 2005). As noted above, rooting depth is a phene aggregate influenced by rooting angle, number, and total metabolic allocation to the root system, so diversity for rooting depth among species influencing productivity represents phene × phene × species interactions. A suite of functional traits associated with acquiring nitrogen in nitrogen-limited grassland plants was proposed which included high carbon:nitrogen tissue, slow metabolic rates, and large root length (Craine et al., 2002). McCormack et al. (2012) found relationships across 12 tree species among root morphology, root chemistry, root lifespan, and whole plant traits, though in another study no clear relationship between root traits such as root diameter and nitrogen concentration was identified (Chen et al., 2013). These studies in natural systems demonstrate a growing awareness of the identification of a root economic spectrum that would be a useful tool for understanding variation in root systems. However, to our knowledge, examples are lacking demonstrating the interactions of specific root phenes for specific functions in natural systems. Most studies rely on interspecific diversity to create root phene variation, which confounds specific phenes with many other covarying factors. Below, we will discuss general approaches to study root phenes and root phene integration that can be extended to any study system.

### GAPS IDENTIFIED BY COMPARING KNOWN INTERACTIONS TO POSSIBLE INTERACTIONS

These case studies demonstrate progress in understanding root phene integration. Most of the studies have been conducted with simulation modeling so must be confirmed empirically, but the work of Miguel (2012) with basal root angle and root hairs is a notable exception where root phene state synergisms were demonstrated in agricultural fields. There are no examples of interactions where resource acquisition phenes affecting metabolic economy, such as axial root number and lateral branching, have been simultaneously manipulated, though Walk et al. (2006) showed an interaction between ARN and respiration mediated through architectural tradeoffs with lateral roots of basal and tap roots. Foraging phenes that influence metabolism may have only additive, or even antagonistic, interactions because of tradeoffs in metabolic economy (Hypothesis 3). Further work is also needed to understand how phenes integrate within and between functional modules.

### APPROACHES FOR STUDYING PHENE INTEGRATION

Many studies analyzing plant traits have relied on comparisons between species for phene state variation and in natural environmental gradients for differences in abiotic conditions. However, such comparisons are confounded by the multitude of differences that exist among species and environments. The use of structured genetic populations that vary for specific phenes but share a common genetic background, evaluated in environments in which specific stresses are imposed, is a more powerful approach when possible (Lynch, 2011). This strategy has the advantage of allowing the comparison of different phene states within a common genetic and phenotypic background, which is especially important given our lack of understanding of phene integration. Populations of RILs have been used both for genetic mapping and for near-isophenic comparisons in common bean and maize (Yan et al., 2004; Ho et al., 2005; Zhu et al., 2005a, b; Ochoa et al., 2006; Zhu et al., 2006; Henry et al., 2010). Near-isophenic lines refer to lines that differ primarily in the state of a single phene, or at least a small number of phenes. Populations of near-isophenic lines may also contain plants with combinations of phene states that allow the study of phene integration. Single gene mutants may not always be useful for studies of phenes because many phenes of interest are controlled by several QTL or genes (Lynch, 2011). While biparental RIL populations are useful for these phenotypic contrasts, their limited diversity (descending from two parents) may not allow the measurement of the breadth of the root phenome. Diversity panels representing broader variation in crops are now being used to probe the breadth of the root phenome. High-throughput phenotyping must increase in extent and intensity (Houle et al., 2010). Extensive phenotyping is accomplished through the sampling of larger numbers of plants of greater diversity. Intensive phenotyping is the measurement of more traits for each sample. Extensive and intensive phenotyping are benefitting from the application of remote sensing, image analysis, and robotics (Fiorani and Schurr, 2013), including with roots (Galkovskyi et al., 2012). Intensity will be further increased by the inclusion of function-valued traits, or phenes that are best described as mathematic functions rather than single values (Kingsolver et al., 2001). Both extensive and intensive phenotyping will contribute to plant phenomics and the study of root phene integration.

Plant phenomics is generating vast amounts of data, and increases in the extent and intensity of phenotyping will accelerate the pace of data collection. The creation and use of data repositories by teams of scientists is imperative. In order for this data to be useful, it must include metadata (higher level information that describes the data and its context). Metadata has the benefits of increasing data longevity and recycling by the creator and others (Michener, 2006). Metadata for functional–structural phenomics must include ontologies for identifying plant structures and research context (Ilic et al., 2007; Madin et al., 2008). Root functional phenomics should include ontologies for roots that represent their phylogeny, genetics, and development (Zobel, 2011), but also their function. Root phenomics will not mature without thorough documentation and sharing of data, especially due to the significant financial costs of root phenotyping.

Rasmusson (1987) proposed developing a “germplasm bank of ideotype traits” where breeders would agree to cooperate to introgress phenes of interest into elite genetic backgrounds. Diversity in crop species traits is often found in landraces or other unimproved varieties (Bayuelo-Jiménez et al., 2011). Recently, Burton et al. (2013a, b) reported substantial variation among RILs, maize landraces and teosintes for both root architectural and root anatomical phenes that could be of use in maize breeding. However, these unimproved genetic backgrounds act as barriers to the inclusion of phenes that comprise a desired ideotype for breeding programs. A collaborative network of plant physiologists and breeders working to identify and understand phenes useful for crop performance would benefit from germplasm banks containing phene states in common genetic backgrounds. In order for researchers and breeders to be able to choose appropriate material for their programs, integration of phenomic and germplasm bank databases will be required. Greater collections of such plant material and relevant genetic resources are available for crop species than for wild plants, but model systems such as *Arabidopsis* and *Populus* may act as bridges for the induction of similar studies in other wild species.

Functional–structural plant modeling is an invaluable tool for the study of root phene integration. *SimRoot *will continue to be of great utility in this endeavor, as will other root simulations such as RootMap (Diggle, 1988; Dunbabin, 2007) and R-SWMS (Javaux et al., 2008). Simulations allow the exploration of trait function beyond what is possible in greenhouse and field studies. Genetic and physiological constraints may make it difficult or impossible to study some phene state combinations, but they can still be modeled. Simulations also allow many different climates, soil types, and nutrient levels to be studied. While only contrasting and extreme phene states may be combined factorially for study in the field or greenhouse due to space and labor limitations, modeling allows a greater phenotypic range and phene combinations to be studied. In an iterative fashion, simulations help focus empirical experimentation on the most interesting phenes and phene interactions, while data from empirical studies parameterize and refine root models (Wullschleger et al., 1994). A recent review of three-dimensional root models highlights the various models’ strengths and weaknesses, and proposes how to advance the field by encouraging wider adoption of root models and by making models more realistic through the inclusion of more explicit plant regulatory networks and soil microorganisms (Dunbabin et al., 2013). Simulations should be integrated with phenomic databases to predict functional implications of phenotypic variation, just as models of predicted gene function and subcellular protein targeting augment genomic databases.

## FUTURE PROSPECTS

The understanding of phenotypic integration requires research comparing multiple states of single phenes in isolation and in combination, generating phene-function landscapes for multiple environments. Understanding the interaction of phenes is particularly important because there may be emergent properties that cannot be predicted from their function in a single phenotypic background. The phenome is the interface of the genome and the environment. Phenes and phenotypes arise through plant development under genetic control as influenced by the environment, so genetic information is useful in understanding phenotypic variation. At the same time, we need to know how phenes influence plant function in specific environments, which will require the collaboration of plant biologists, soil scientists, and climatologists. Many phenes will not be under single gene control, so the use of single gene mutants for phene studies may limit inquiry to the presence or absence of a particular phene, but we also need to know how variation in phene states contributes to different aspects of plant function. The use of emerging technologies in plant genetics, such as RNA interference, may allow more complex developmental manipulation through changes in expression levels of several genes that could possibly give rise to ranges of phene states in common genetic and phenotypic backgrounds (Katoch and Thakur, 2013).

Phenes are properties of the organism that have been neglected in the genomic era. The organism is the fundamental biological unit of organization for studies of phenes and phene interaction. It is surprising how little research focuses on organisms *per se*, in contrast to the organism being treated primarily as a tool to understand genes or ecosystems. Organisms are the entities on which natural and artificial selection act, which genes influence, and of which ecosystems are composed (Lewontin, 1970). The variation in phenes embodied within a taxon cannot simply be averaged to generate an ideal individual because this variation has functional and evolutionary importance. Progress in understanding the plant genome is stunning, and currently far outstrips our understanding of the plant phenome, despite the fact that the plant phenome is at least as complex as the genome and arguably more important for human welfare.

The study of phenes is hindered by the lack of relevant conceptual frameworks. Here we have discussed phenes in the traditional context as building blocks of an organism’s phenotype. In some cases it may not be clear whether a phene is truly elemental, as it may be influenced by other traits at lower levels of organization. For example, basal root number in common bean was found to be influenced by basal root whorl number (Miguel, 2012). However, the discovery of even more elemental phenes is a useful outcome of applying the phene perspective. The ambiguity of the phene might be necessary for it to be applied in diverse fields and research programs, but the science of the phenome, phenes and phene interactions will be aided by the development of more precise and informative theoretical frameworks. A better understanding of integrated phenotypes would have benefits for other fields of biology and agriculture, such as how natural selection has led to the diversity of forms observed within and among species, and how improved crop varieties can be designed and developed. Trait-based, or ideotype, breeding is an important avenue for crop improvement, and has been shown to be more efficient than yield-based selection in some situations (Annicchiarico and Pecetti, 1998). Yield and metrics closely associated with yield, such as number of grains, may obscure the advantages of phene states that happen to be in otherwise poor backgrounds. Genetic and developmental pathways may overlap among quantitative traits such as root phenes, so genetic associations with yield or other functional responses are also of limited use. Phene utility should be measured in the field, and for specific environmental stresses, because the advantages of some phene states may only reveal themselves when resources are limiting. Understanding the functional utility of specific root phenes and their interactions requires the employment of near-isophenic plant material in the field and simulation modeling. The opportunities created by the ability to understand the fitness landscape of integrated ideotypes will eventually lead to greater understanding of ecosystem structure and function, and to superior crop lines bred for specific agricultural contexts.

Alleviation of world hunger despite a burgeoning human population, continually degrading natural resources, and global climate change is a primary human challenge for the 21st century. New crop lines with superior soil resource acquisition will be a valuable tool to that end (Lynch, 2007b; Lynch and Brown, 2012). In natural systems, understanding how root phenes influence community structure and ecosystem function will inform policies to manage anthropogenic effects on the climate and environment. Clarification and refinement of phene integration theory, simulation and field studies of phenes and phene interactions, and the distribution of results and plant materials are all essential for the success of this unprecedented opportunity to deploy phenes to provide solutions for pressing world problems.

## Conflict of Interest Statement

The authors declare that the research was conducted in the absence of any commercial or financial relationships that could be construed as a potential conflict of interest.
